# HIV-1 Vpu Interference with Innate Cell-mediated Immune Mechanisms

**DOI:** 10.2174/157016212800792513

**Published:** 2012-06

**Authors:** Johan K Sandberg, Sofia K Andersson, Susanna M Bächle, Douglas F Nixon, Markus Moll

**Affiliations:** 1 Center for Infectious Medicine, Department of Medicine, Karolinska Institutet, Karolinska University Hospital Huddinge, Stockholm, Sweden; 2 Division of Experimental Medicine, Department of Medicine, University of California, San Francisco, San Francisco, CA, USA

**Keywords:** HIV-1, iNKT cells, CD1d, Vpu, NK cells, immune evasion, NTB-A, Nef, Vpr.

## Abstract

The HIV-1 accessory protein Vpu is emerging as a viral factor with a range of activities devoted to counteracting host innate immunity. Here, we review recent findings concerning the role of Vpu in hampering activation of cellular immune responses mediated by CD1d-restricted invariant natural killer T (iNKT) cells and natural killer (NK) cells. The two key findings are that Vpu interferes with CD1d expression and antigen presentation, and also with expression of the NK cell activation ligand NK-T and B cell antigen (NTB-A). Both these activities are mechanistically distinct from CD4 and Tetherin (BST-2) down-modulation. We summarize the mechanistic insights gained into Vpu interference with CD1d and NTB-A, as well as important challenges going forward, and discuss these mechanisms in the context of the role that iNKT and NK cells play in HIV-1 immunity and immunopathogenesis.

## INTRODUCTION

Cellular immunity is important for host defense against viral infections. Immune cells with innate characteristics have the capacity to respond rapidly without the need for an extensive priming phase, and such cells may therefore be particularly important in controlling viral replication during the first days of an infection. This group of cells includes invariant CD1d-restricted natural killer T (iNKT) and natural killer (NK) cells. iNKT cells are characterized by a semi-invariant T cell receptor (TCR) that uses the TCR α-chain segments Vα24 and Jα18 preferentially paired with the variable β-chain Vβ11 segment [1]. They have a surface phenotype reminiscent of effector memory T cells [2, 3], and can be subdivided into CD4+ and CD4- subsets with distinct functional profiles [4, 5]. iNKT cells respond rapidly to two distinct types of stimuli. First, they can recognize foreign glycolipid antigens presented by CD1d molecules expressed by antigen presenting cells such as dendritic cells (DCs), monocytes and B cells [6, 7]. This mode of activation has been observed with antigens from certain species of bacteria, such as *Sphingomonas* [8, 9], and *Borrelia burgdorferi* [10]. Secondly, iNKT cells can be activated in the absence of cognate CD1d-presented antigen through TCR recognition of endogenous lipids in the context of inflammatory cytokines such as IL-12 and IL-18 [11, 12]. The endogenous antigens recognized in this way have long remained elusive, but very recently β-D-glucopyranosylceramide (β-GlcCer) was identified as one such antigen [13]. β-GlcCer was found to accumulate in response to infection as well as TLR agonists, suggesting that this type of recognition may be relevant for several pathogens including viruses.

NK cells mediate their effector function through cytolysis and production of cytokines and chemokines, and these activities are regulated by a vast array of activating and inhibitory receptors [14, 15]. One important activating receptor is natural-killer group 2 member D (NKG2D) which mediates activation upon engagement of stress-induced MHC class I-related chain A and B (MICA/B) and members of the UL16-binding protein (ULBP) family [16, 17]. Another activating NK cell receptor is DNAX accessory molecule-1 (DNAM-1), which recognizes both the poliovirus receptor (PVR) (CD155) and Nectin-2 (CD112) [18]. During a viral infection, the activating and inhibitory receptors collectively have the delicate task to allow and facilitate activation of anti-viral effector mechanisms against infected cells, while at the same time limiting collateral damage to uninfected host cells [19]. Convincing evidence for an important role of NK cells in viral infections in humans comes from patients with NK cell defects that have higher susceptibility to certain viruses, including herpes viruses [20-22]. There is also evidence that several viruses modulate expression of ligands for activating and inhibitory NK cell receptors to evade recognition by these cells [22].

In this paper, we briefly review what is currently known about the role of iNKT cells and NK cells in HIV-1 infection with a special focus on the recent findings that the HIV-1 *vpu* gene-product interferes with expression of the iNKT cell ligand CD1d [23], as well as the homotypic activating NK cell receptor/ligand NTB-A [24].

## iNKT CELLS IN HIV-1 INFECTION

**Fig. (1):**
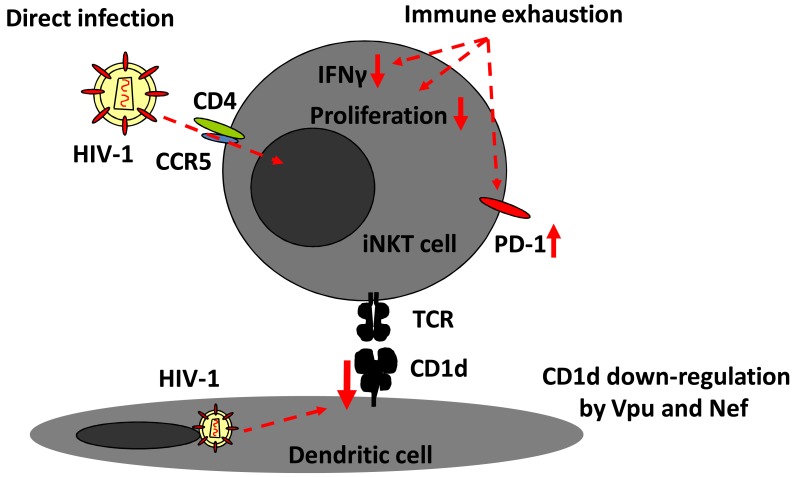
**HIV-1 impacts iNKT cells at three levels.** Figure illustrates the three known ways that HIV-1 infection interferes with the function of iNKT cells. iNKT cells are susceptible to direct HIV-1 infection. The residual iNKT cells that persist in chronic infection are functionally impaired and display signs of exhaustion, with poor IFNγ production, low proliferative capacity, and frequent PD-1 expression. Finally, HIV-1 carries two independent mechanisms to interfere with CD1d-mediated antigen presentation via the action of the Vpu and Nef proteins.

In 2002, three groups independently reported that levels of iNKT cells in peripheral blood are severely depressed in HIV-1 infected adults [25, 26], and children [27] (Fig. **1**). This was subsequently confirmed by others [28], and similar findings were made in non-human primate models of SIV infection [29]. The loss of iNKT cells appears to be rapid in many patients, and this may be at least partly due to high levels of CCR5 expression making them preferential targets for HIV-1 (Fig. **1**) [26, 27, 30]. Somewhat conflicting data exist regarding the ability of antiretroviral treatment (ART) to rescue quantitative and qualitative aspects of the iNKT cell compartment, and the efficacy of ART in this context may depend on timing of initiation of treatment [28, 31-34]. Some patients retain almost normal numbers of iNKT cells throughout untreated chronic HIV-1 infection, but those cells express an exhausted phenotype with elevated PD-1 expression [32, 35]. The mechanisms by which iNKT cells respond to HIV-1 infection are incompletely known, although iNKT cell supernatants have been shown to inhibit HIV replication *in vitro* [28]. Solid, albeit indirect, evidence for an important role of iNKT cells in immune defense against HIV-1 comes from the recent observations that the virus carries several mechanisms for down-regulation of the iNKT cell ligand CD1d (Fig. **1**) [23, 36, 37].

## INTERFERENCE WITH CD1d-MEDIATED ANTIGEN PRESENTATION BY HIV-1 VPU AND NEF

Over the last decade is has become clear that recognition of lipid antigens by the immune system plays an important role in the host defense to infectious diseases [38]. In humans, endogenous and exogenous lipid-antigens are presented to T cells by four different CD1 molecules; CD1a, CD1b and CD1c (group I), and CD1d (group II) [39, 40]. These four types of CD1 molecules have distinct cellular expression profiles as well as different sub-cellular distributions, indicating a sophisticated system to survey the presence of lipid antigens [39, 40]. Expression of group I CD1 molecules is mainly confined to professional antigen presenting cells (APCs) such as DCs and Langerhans cells, while CD1d is also expressed in monocytes, macrophages, B cell subsets, and some non-hematopoietic cells [7]. Noteworthy, CD1d is absent from the surface of naïve T cells but can be expressed upon T cell activation [41]. Thus, CD1 molecules have an expression pattern that largely overlaps with the host cell range of HIV-1.

**Fig. (2):**
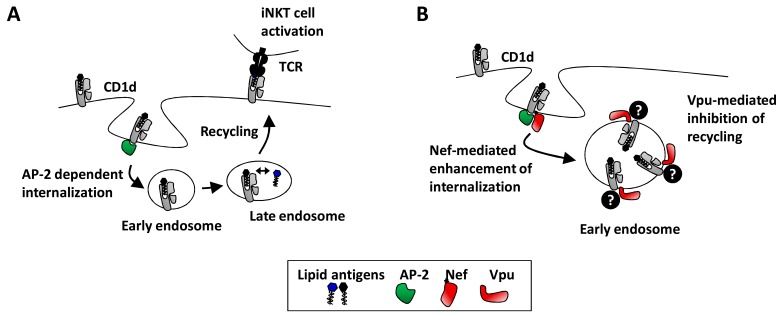
**HIV-1 Vpu and Nef interfere with CD1d-mediated antigen presentation.** **A**) The normal AP-2 dependent trafficking of CD1d through early and late endosomal compartments for antigen loading and presentation to iNKT cells. **B**) In HIV-1 infected cells this process is disrupted by Nef that enhances internalization, and by Vpu that inhibits recycling of CD1d back to the cell surface. Possible co-factors in the Vpu-mediated inhibition of recycling remain to be identified.

In recent years it has become evident, that the two accessory HIV-1 proteins Nef and Vpu inhibit the surface expression of CD1d in a concerted action by intervening with the intracellular trafficking of CD1d. Under normal conditions, CD1d molecules, after initial trafficking to the cell surface, constitutively recycle between the surface and endosomal compartments to survey the endocytic system for the presence of lipid antigens [42]. Nef tends to retain CD1d in the trans-Golgi network and increases its rate of internalization from the cell surface [36]. Vpu on the other hand decreases the recycling of CD1d from endosomal compartments to the cell surface, and co-localization of Vpu and CD1d in early endosome antigen 1 (EEA-1) positive structures indicates retention of CD1d in the early endosome [23]. Putting these observations together, we suggest a model where Nef and Vpu in a synergistic fashion achieve the efficient inhibition of CD1d cell surface expression in productively infected DCs (Fig. **2**). This model is supported by the finding that virus mutants lacking the expression of either Nef or Vpu still partially down-regulate CD1d whereas a Nef/Vpu double-defective virus completely lacks this activity [23]. Whereas the detailed molecular mechanisms and structural requirements remain to be elucidated, physical interaction between CD1d and Nef as well as CD1d and Vpu has been detected [23, 37]. It is, however, unclear if the observed interactions are of direct nature or if other cellular co-factors are involved in complex formation and down-regulation. In addition to the effects of Nef and Vpu, a single report suggested that adding soluble HIV gp120 protein to U937 cells reduced surface CD1d expression on these cells [43]. The potential mechanisms involved and relationship to the Vpu- and Nef-mediated effects on the CD1d antigen presentation pathway remains to be determined.

CD1d internalization from the cell surface requires interaction of a tyrosine-based motif in its cytoplasmic tail with adaptor protein complex-2 (AP-2) [42]. Mutation of the known AP-2 binding sites in Nef strongly affected CD1d down-regulation, indicating that Nef enhances CD1d internalization by subverting the regular AP-2 mediated trafficking of CD1d [36, 44]. Although Vpu contains three potential AP-binding motifs in its cytoplasmic domain, Vpu interaction with AP-2 or other adaptor protein complexes has so far not been demonstrated [45], making involvement of AP-2 in Vpu-mediated inhibition of CD1d expression unlikely. Down-regulation of CD4 and tetherin involves association of Vpu with the cellular co-factor β-TrCP to mediate ubiquitination and proteasomal degradation, and phosphorylation of Vpu at serine residues 52 and 56 may be important in this process [45]. The contributions of proteasomal and lysosomal degradation in down-regulation of tetherin from the cell surface are, however, controversially discussed [46, 47]. Moreover, enhancement of virus particle release by Vpu may occur in the complete absence of tetherin degradation suggesting that the two processes are not related [48]. However, because CD1d down-regulation from the cell surface does not require Vpu phosphorylation (our unpublished observation), involvement of β-TrCP-mediated ubiquitination and proteasomal degradation is unlikely to be involved in this process. These results collectively prompt the search for cellular co-factors binding Vpu and contributing to the Vpu-mediated retention of CD1d in early endosomal compartments.

The consequences of CD1d down-regulation have been assessed in different experimental systems and comprise early and late events of iNKT cell activation. HIV-1 infected DCs, as well as Vpu-transfected cell lines, loaded with the model lipid antigen α-galactosylceramide have a significantly reduced capacity to induce the formation of an immunological synapse with iNKT cells [23]. Likewise, the activation of iNKT cells is reduced when either Nef- or Vpu-transfected cell lines or HIV-1 infected DCs are used for stimulation, demonstrating the potency of viral interference with CD1d expression [23, 36, 37]. Reversion of the phenotype by deleting functional expression of Nef and Vpu fully restores the capacity of DCs to induce iNKT cell activation, indicating that HIV-1 infected DCs are not unable *per se* to activate iNKT cells [23].

Interference of HIV-1 with the group I CD1 system and T cell responses to antigens presented by these CD1 molecules has not been much studied. The effect of HIV-1 on group I CD1 proteins was described in only a single report, where CD1a down-regulation from the cell surface was observed in DCs infected with recombinant HIV-1 pseudotyped with VSV-G [49]. In that study, expression of CD1b and CD1c was unaffected, and even down-regulation of CD1d was not observed. Mechanistically, down-regulation of CD1a appeared to be linked to the redistribution of this molecule from the cell surface to LAMP-1 positive compartments, an effect mediated by the Nef protein. The biological consequences of HIV-1 interference with CD1a expression remain unclear and need further investigation.

## NK CELLS IN HIV-1 INFECTION

NK cells play a very important and multifaceted role in control of HIV-1 infection and replication [50]. NK cells can fight HIV-1 *via *direct cytolysis of infected cells [51-53], and production of chemokines and cytokines that directly or indirectly inhibit viral entry and replication [54, 55]. In addition, HIV-binding antibodies allow NK cells to mediate antibody-dependent cellular cytotoxicity (ADCC), which probably also contributes to control of virus [56, 57].

NK cell activity is balanced by signals from activating and inhibitory receptors [14, 15]. Killer-cell immunoglobulin-like receptors (KIRs) and their HLA class ligands are of particular interest in the HIV field, since the observation that individuals expressing the activating receptor KIR3DS1 together with certain HLA-Bw4 alleles show slower progression to AIDS than people lacking either one or both of these receptors [58]. In addition, individuals carrying some alleles of the inhibitory KIR3DL1 receptor also display slower HIV disease progression [59]. The importance of the KIR3DL1/DS1 interaction with HLA-Bw4 has been substantiated by the observation that KIR3DS1+ NK cells are more effective in killing HIV-infected HLA-Bw4+ target cells [60]. Furthermore, KIR3DL1/DS1+ NK cells expand in infected subjects carrying HLA-Bw4 [61, 62], and this NK cell expansion correlates with viral load in chronic infection [62].

Engagement of the NK cell compartment in the response to HIV-1 furthermore results in global changes in receptor expression levels and subset distribution [63, 64]. These changes include the relative subset redistribution with fewer CD56dim NK cells and expansion of an aberrant CD56 negative NK cell subset. The detailed mechanisms underlying these changes in receptor expression and subset composition of NK cells still remain largely unknown.

## VPU INTERFERENCE WITH NK CELL CYTOLYSIS

**Fig. (3):**
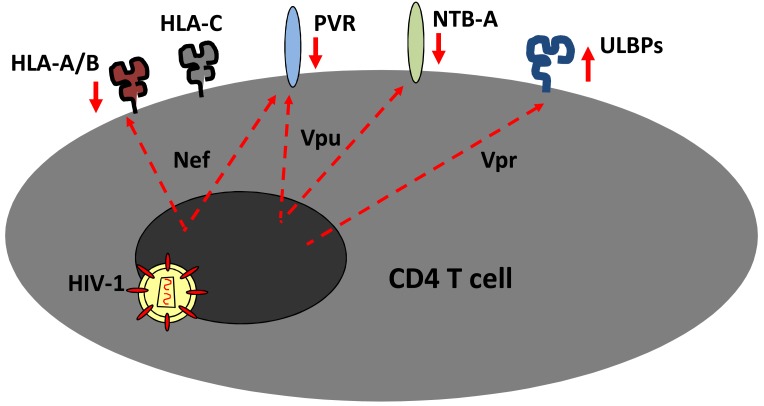
**Vpu, Nef and Vpr modulate expression of NK cell ligands on the surface of HIV-1 infected cells.** Cell surface ligands of NK cell receptors are modulated by HIV-1 accessory proteins Nef, Vpr and Vpu resulting in the down-regulation of HLA-A and -B molecules, up-regulation of the ULBP NKG2D ligands, inhibition of NTB-A expression, and down-regulation of the DNAM-1 ligand PVR.

With the mounting evidence that NK cells have an important role in the control of HIV-1 infection, one may speculate that the virus should have evolved means to evade NK cell recognition and killing of infected cells. This indeed seems to be the case. It was recently discovered that the NK-T and B cell antigen (NTB-A) is down-regulated from the surface of HIV-1 infected cells [65], in a Vpu-dependent manner [24]. NTB-A is a member of the signaling lymphocytic activation molecule (SLAM) family of receptors [66]. It is expressed on all lymphocytes and acts in a homotypic manner as a co-activating receptor in NK cells [24]. Notably, NTB-A triggering is necessary to induce efficient lysis of target cells upon engagement of the activating receptor NKG2D, and Shah *et al*. were able to show that down-regulation of NTB-A from the surface of HIV-1 infected cells contributes to the relatively poor NK cell cytolytic activity against such cells (Fig. **3**) [24]. Similar to Vpu-mediated down-regulation of CD1d, NTB-A down-regulation does not involve β-TrCP recruitment and subsequent degradation of the protein, nor the enhanced internalization from the cell surface. Interestingly, Vpu mediates its effect on NTB-A *via *its transmembrane region, and leads to accumulation of the protein in an as of yet not described intracellular compartment.

HIV-1 Nef is known to inhibit expression of HLA-A and -B class I molecules [67], and the *vpr* gene-product induces an increased expression of some activating NKG2D ligands (Fig. **3**) [68-70]. These changes would be expected to make cells tasty targets for NK cell killing. Vpu-mediated interference with NTB-A expression may thus be very important for HIV-1 to evade elimination by NK cells. Furthermore, it was recently observed that Nef and Vpu together act to down-regulate the DNAM-1 ligand PVR from the surface of infected cells [71] (Fig. **3**). Nef appears to mediate this effect on PVR by a mechanism similar to that used to down-regulate MHC class I molecules, whereas the mechanism underlying Vpu’s effects on PVR is unknown [71].

Yet another layer of complexity comes from the observation that NKG2D functions as an activating and co-activating receptor in T cells, including iNKT cells [72]. Vpr-induced expression of ULBPs may thus to some extent counteract the activities by Vpu to inhibit activation of both NK cells and iNKT cells. When viewed together with the effects on tetherin and CD1d, the findings concerning down-regulation of ligands for activating NK cell receptors indicate that Vpu may be a critical factor for HIV-1 evasion from innate immune mechanisms [73].

## FUTURE PERSPECTIVES

Significant effort has been put into investigating the capacity of Vpu proteins derived from the different HIV-1 groups to interfere with surface expression and function of CD4 and Tetherin. Whereas the Vpu proteins of pandemic group M viruses are able to induce both CD4 degradation and antagonize Tetherin, HIV-1 group N, O and P Vpu proteins perform only one of these functions [74, 75]. The non-pandemic groups of viruses are geographically restricted to West-central Africa, and acquisition of a functionally fully competent Vpu may have contributed to the global success of HIV-1 group M viruses [76]. In this context it will be important to investigate if the capacity to inhibit expression of CD1d and NTB-A is conserved among Vpu proteins derived from the different HIV-1 groups.

The question arises why HIV-1 with its limited genome, in the face of severe selection pressures, maintains the ability to interfere with the lipid antigen presenting molecule CD1d. To our knowledge, neither HIV-specific T cells restricted by CD1 molecules nor HIV-derived lipid antigens have yet been identified. Furthermore, viruses do not encode lipids, and the lipid envelope of HIV is derived from host cell membranes. The need to interfere with CD1-mediated antigen presentation is therefore not self-evident. A possible scenario is that iNKT cell recognition of infected cells is independent of foreign lipid antigen. Cytokines produced by a virus infected or exposed cell in combination with the recognition of endogenous lipid antigens presented by CD1d may be sufficient for iNKT cell activation. It is also possible that an altered lipid metabolism in HIV infected cells may generate an altered endogenous lipid antigen content in CD1 molecules. Furthermore, the recent discovery that CD1a and CD1c are able to present lipid-modified peptides opens up the possibility that virally derived lipopeptides may be presented by CD1 molecules [77, 78]. Future studies may reveal if the myristoylated HIV-1 proteins p17 and Nef give rise to CD1-presented lipopeptide structures enabling T cells to recognize HIV-1 infected cells.

Nef is expressed early, whereas Vpu is expressed late in the HIV-1 life cycle [79]. The effects that these viral proteins have on CD1d, NTB-A, MHC class I, and PVR may therefore vary over the life span of a productively infected cell. Detailed studies of the expression of these viral factors as well as their target host proteins over time in DCs and CD4 T cells may give important insight into the complexity of Vpu- and Nef-mediated evasion from innate cell-mediated immunity.

Elucidation of the fine mechanistic detail of Vpu-mediated inhibition of CD1d and NTB-A expression will be important for the basic understanding of these apparent immune evasion mechanisms. Furthermore, this level of understanding may allow rational design of a novel class of anti-viral treatments. We speculate that pharmaceutical interference with these functions of Vpu might help improve the contribution of innate immunity to control of viral replication.
